# Integrating psychiatry and family medicine in the management of somatic symptom disorders: Diagnosis, collaboration, and communication strategies

**DOI:** 10.1002/jgf2.725

**Published:** 2024-10-28

**Authors:** Victor Ajluni

**Affiliations:** ^1^ Wayne State University Livonia Michigan USA

**Keywords:** anxiety, behavioural science, depression, family medicine, hospital general medicine, insomnia

## Abstract

Somatic symptom disorders (SSDs) present a complex interplay of physical and psychological factors, necessitating an integrative approach to diagnosis and management. This article explores the collaborative efforts between family medicine and psychiatry in addressing SSDs, emphasizing the importance of a multidisciplinary strategy for comprehensive patient care. Effective diagnosis involves recognizing the significance of both somatic symptoms and the patient's psychological response, with tools like structured clinical interviews and self‐report questionnaires playing crucial roles. Management strategies include psychotherapeutic interventions such as cognitive behavioral therapy (CBT), pharmacological treatments, and lifestyle modifications, all tailored to the patient's needs. Communication strategies are vital in validating patients' experiences while addressing underlying psychiatric issues. Techniques such as active listening, biopsychosocial framing, and the teach‐back method foster trust and improve treatment adherence. Cultural considerations and the use of interpreters enhance communication with diverse patient populations. Training programs for healthcare providers further improve competency in managing SSDs. This integrative approach aims to enhance patient outcomes by addressing the multifaceted nature of SSDs through collaborative care, effective communication, and comprehensive treatment planning.

## Graphical abstract

Somatic symptom disorders (SSDs) present a complex challenge in modern healthcare, requiring a nuanced approach that bridges the gap between physical and psychological medicine. This review explores the current understanding of SSDs, focusing on diagnostic challenges, management strategies, and the crucial role of interdisciplinary collaboration between family medicine and psychiatry. Effective management of SSDs involves a multifaceted approach, combining psychotherapeutic interventions, judicious use of pharmacological treatments, and skilled communication strategies that validate patients‘ experiences while addressing underlying psychiatric issues.

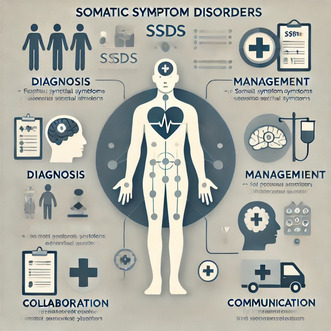

## 1 INTRODUCTION

Somatic symptom disorders (SSDs) represent a complex intersection of physical and psychological experiences, challenging both patients and healthcare providers in their diagnosis and management. These disorders, characterized by persistent physical symptoms that disrupt daily life and are associated with excessive thoughts, feelings, or behaviors, have historically been a source of debate and difficulty in medical practice.^1^ The intricate interplay between bodily sensations and psychological processes in SSDs necessitates a multidisciplinary approach, particularly involving collaboration between family medicine and psychiatry.^2^

This review aims to explore the current understanding of somatic symptom disorders, focusing on diagnostic challenges, management strategies, and the crucial role of interdisciplinary collaboration. Furthermore, it will examine effective communication strategies that validate patients' experiences while addressing underlying psychiatric issues. By synthesizing recent research and clinical guidelines, this paper seeks to provide a comprehensive overview of the state of knowledge in this field and highlight areas for future investigation and improvement in patient care.

The significance of this topic is underscored by the prevalence of SSDs, with estimates suggesting they affect up to 5%–7% of the general population.^3^ These disorders not only cause significant distress and impairment for individuals but also place a substantial burden on healthcare systems, often resulting in frequent medical consultations and investigations.^4^ As such, enhancing our approach to SSDs has the potential to improve patient outcomes and optimize healthcare resource utilization.

## 2 DIAGNOSIS OF SOMATIC SYMPTOM DISORDERS

The diagnosis of somatic symptom disorders (SSDs) presents unique challenges due to the complex interplay between physical symptoms and psychological factors. The Diagnostic and Statistical Manual of Mental Disorders, Fifth Edition (DSM‐5), marked a significant shift in the conceptualization of these disorders, moving away from the emphasis on medically unexplained symptoms to focus on the patient's thoughts, feelings, and behaviors in response to their symptoms.^5^

### 2.1 Key diagnostic criteria

According to the DSM‐5, the diagnosis of SSD requires:

1. One or more somatic symptoms that are distressing or result in significant disruption of daily life.
2. Excessive thoughts, feelings, or behaviors related to the somatic symptoms or associated health concerns.
3. Persistent symptom state (typically more than 6 months).^6^

This revised approach acknowledges that somatic symptoms can coexist with diagnosed medical conditions, shifting the focus to the patient's response to their symptoms rather than the etiology of the symptoms themselves.^7^

### 2.2 Diagnostic challenges

Several factors complicate the diagnostic process for SSDs:

1. Overlap with medical conditions: Distinguishing between SSDs and organic pathology can be challenging, particularly when symptoms are nonspecific or fluctuating.^8^
2. Comorbidity: SSDs often co‐occur with other psychiatric disorders, such as depression and anxiety, which can mask or exacerbate somatic symptoms.^9^
3. Cultural variations: Cultural factors significantly influence the expression and interpretation of somatic symptoms, necessitating culturally sensitive diagnostic approaches.^10^
4. Stigma: The perceived stigma associated with psychiatric diagnoses may lead patients to emphasize physical symptoms over psychological distress.^11^

### 2.3 Diagnostic tools and approaches

To address these challenges, several diagnostic tools and approaches have been developed:

1. Structured clinical interviews: Tools like the Structured Clinical Interview for DSM‐5 (SCID‐5) can help ensure a comprehensive assessment of symptoms and their impact.^12^
2. Self‐report questionnaires: Instruments such as the Patient Health Questionnaire‐15 (PHQ‐15) and the Somatic Symptom Scale‐8 (SSS‐8) can aid in screening and monitoring symptom severity.^13^
3. Collaborative assessment: Involving both primary care physicians and mental health professionals in the diagnostic process can provide a more holistic evaluation of the patient's condition.^14^
4. Longitudinal assessment: Given the persistent nature of SSDs, diagnosis often requires ongoing evaluation over time to distinguish from transient somatic complaints.^15^

The accurate diagnosis of SSDs is crucial for appropriate management and treatment planning. It requires a careful balance between acknowledging the reality of physical symptoms and recognizing the psychological factors that influence their presentation and impact. As our understanding of the mind–body connection continues to evolve, so too must our diagnostic approaches to these complex disorders.

## 3 MANAGEMENT STRATEGIES FOR SOMATIC SYMPTOM DISORDERS

The management of somatic symptom disorders (SSDs) requires a multifaceted approach that addresses both physical symptoms and psychological factors. Effective treatment strategies often involve a combination of psychotherapeutic interventions, pharmacological treatments, and lifestyle modifications.

Psychotherapeutic interventions are central to the management of SSDs. Cognitive Behavioral Therapy (CBT) has demonstrated significant efficacy in treating SSDs.^16^ It focuses on identifying and challenging maladaptive thoughts about symptoms, developing coping strategies for symptom management, and reducing avoidance behaviors while promoting gradual exposure to feared situations. Mindfulness‐based therapies help patients develop a non‐judgmental awareness of their bodily sensations, reducing symptom‐related anxiety and improving overall well‐being.^17^ Acceptance and commitment therapy (ACT) encourages patients to accept their symptoms while committing to valued life activities, potentially reducing the impact of symptoms on daily functioning.^18^

While no medications are specifically approved for SSDs, certain pharmacological interventions may be beneficial. Antidepressants, such as selective serotonin reuptake inhibitors (SSRIs) and serotonin‐norepinephrine reuptake inhibitors (SNRIs), can help manage comorbid depression and anxiety, and may also have direct effects on somatic symptoms.^19^ In some cases, atypical antipsychotics at low doses may be considered for severe, treatment‐resistant cases, although their use remains controversial.^20^ For patients with prominent pain symptoms, medications such as pregabalin or gabapentin may be considered.^21^

Lifestyle modifications and complementary approaches can also play a significant role in managing SSDs. Regular physical activity can improve mood, reduce stress, and alleviate certain somatic symptoms.^22^ Stress reduction techniques, such as progressive muscle relaxation, biofeedback, and yoga, may help manage stress‐related exacerbations of symptoms.^23^ Improving sleep quality through consistent sleep schedules and sleep hygiene practices can positively impact both physical and psychological well‐being.^24^

Given the complex nature of SSDs, integrated care models have shown promise. Collaborative care models, involving primary care physicians, mental health professionals, and care coordinators, have demonstrated improved outcomes and cost‐effectiveness.^25^ The stepped care approach involves starting with low‐intensity interventions and progressively increasing treatment intensity based on patient response and needs.^26^ Providing clear, non‐judgmental information about the nature of SSDs can improve treatment adherence and outcomes.^27^

Several challenges persist in the management of SSDs. Some patients may show limited response to standard interventions, necessitating personalized and persistent treatment approaches.^28^ Excessive medical investigations or treatments can reinforce illness behaviors and lead to iatrogenic complications.^29^ Maintaining a therapeutic alliance can be challenging, particularly when patients feel their symptoms are being dismissed or psychologized.^30^

Effective management of SSDs requires a patient‐centered, multidisciplinary approach that addresses both the physical and psychological aspects of the disorder. By combining evidence‐based treatments with compassionate care, healthcare providers can help patients improve their quality of life and functional status.

## 4 COLLABORATION BETWEEN FAMILY MEDICINE AND PSYCHIATRY

The complex nature of somatic symptom disorders (SSDs) necessitates a collaborative approach between family medicine and psychiatry. This interdisciplinary cooperation is crucial for providing comprehensive care that addresses both the physical and psychological aspects of these disorders.

Collaboration between family physicians and psychiatrists is important for holistic patient care, as they bring complementary expertise, allowing for a more complete understanding of the patient's condition.^31^ Early detection and intervention can be facilitated through collaboration, potentially preventing chronic illness trajectories.^32^ Moreover, coordinated care can optimize resources by reducing unnecessary medical investigations and treatments, leading to more efficient use of healthcare resources.^33^

In the consultation‐liaison psychiatry model, psychiatrists provide consultations within primary care settings, offering expertise in the diagnosis and management of SSDs.^34^ Integrated care teams, which include family physicians, psychiatrists, psychologists, and other healthcare professionals, work together to provide comprehensive care.^35^ Shared care models involve family physicians and psychiatrists sharing responsibility for patient care, with regular communication and joint decision‐making.^36^ Telepsychiatry can also facilitate collaboration between family physicians and remote psychiatric consultants in areas with limited access to psychiatric services.^37^

Key areas of collaboration include diagnostic clarification, where joint assessments can help differentiate SSDs from other medical and psychiatric conditions.^38^ Collaborative decision‐making can lead to more comprehensive and tailored treatment plans.^39^ Coordinated medication reviews can minimize polypharmacy and potential drug interactions.^40^ A unified approach to patient education can reinforce key messages and improve treatment adherence.^41^ Regular communication between providers can facilitate early detection of changes in the patient's condition or treatment response.^42^

However, challenges in collaboration include time constraints, as busy clinical schedules can make regular communication between providers challenging.^43^ Different practice cultures between family medicine and psychiatry may need to be bridged for effective collaboration.^44^ Ensuring secure and efficient sharing of patient information while maintaining confidentiality can be complex.^45^ Additionally, some healthcare systems may not adequately reimburse collaborative care models, creating financial disincentives.^46^

Strategies to enhance collaboration involve joint training programs that foster mutual understanding and collaboration skills.^47^ Establishing standardized communication protocols can improve collaboration efficiency.^48^ Shared electronic health records can facilitate information sharing and coordinated care.^49^ Regular case conferences provide opportunities for collaborative problem‐solving.^50^ Collaborative care coordinators can facilitate communication and ensure continuity of care across disciplines.^51^

The collaboration between family medicine and psychiatry is essential for providing optimal care to patients with SSDs. By leveraging the strengths of both disciplines, healthcare providers can offer more comprehensive, patient‐centered care that addresses the complex interplay of physical and psychological factors in these disorders.

## 5 COMMUNICATION STRATEGIES

Effective communication is crucial in the management of somatic symptom disorders (SSDs). It plays a vital role in validating patients' experiences while addressing underlying psychiatric issues. The following strategies can help healthcare providers navigate these complex interactions:

Validating the patient's experience is essential. Active listening, which involves demonstrating genuine interest and attention to the patient's narrative, can build trust and rapport.^52^ Acknowledging distress by explicitly recognizing the reality and impact of the patient's symptoms is crucial for establishing a therapeutic alliance.^53^ Avoiding dismissive language is also important; refraining from phrases that might be perceived as minimizing or delegitimizing the patient's experience is essential.^54^ Additionally, normalizing the patient's experiences by helping them understand that their experiences are not uncommon can reduce feelings of isolation and stigma.^55^

Addressing underlying psychiatric issues involves presenting a biopsychosocial framing that integrates biological, psychological, and social factors, helping patients understand the multifaceted nature of their symptoms.^56^ Gradually introducing psychological concepts can increase acceptance and reduce resistance.^57^ Emphasizing the mind–body connection by educating patients about the well‐established links between mental and physical health can facilitate acceptance of psychological interventions.^58^ Collaborative goal‐setting, which involves patients in defining treatment goals, can increase engagement and align expectations.^59^

Specific communication techniques can be beneficial. Motivational interviewing, a patient‐centered approach, can help resolve ambivalence and increase motivation for change.^60^ Using metaphors and analogies can help explain complex concepts about SSDs in an accessible way.^61^ The teach‐back method, which involves asking patients to explain their understanding of the information provided, can ensure clear communication and identify areas needing clarification.^62^ Providing written summaries can reinforce key points and serve as a reference for patients.^63^

Addressing challenging situations requires skill and sensitivity. When managing disagreements, acknowledging the patient's perspective while gently presenting alternative viewpoints can be helpful.^64^ Dealing with anger or frustration involves remaining calm, validating emotions, and focusing on shared goals to de‐escalate tense situations.^65^ When addressing requests for unnecessary tests or treatments, explaining the potential risks of excessive medical interventions while offering alternative management strategies can help redirect patient expectations.^66^

Cultural considerations are also important. Cultural humility involves recognizing and respecting cultural differences in symptom expression and health beliefs, which is crucial for effective communication.^67^ Using interpreters when language barriers exist can facilitate accurate and culturally sensitive communication.^68^ Adapting explanatory models to align with the patient's cultural background can improve understanding and acceptance of SSDs.^69^

Training and skill development are key to effective communication. Communication skills training through structured programs focusing on SSD‐specific communication challenges can improve provider competence and confidence.^70^ Reflective practice, which encourages healthcare providers to reflect on their communication experiences, can lead to continuous improvement.^71^ Regular peer feedback and review on communication skills can help identify areas for improvement and share best practices.^72^

Effective communication in the context of SSDs requires a delicate balance between validating patients' physical experiences and addressing underlying psychological factors. By employing these strategies, healthcare providers can foster trust, improve treatment adherence, and ultimately enhance outcomes for patients with SSDs.

## 6 CONCLUSION

The management of somatic symptom disorders (SSDs) presents a significant challenge to modern healthcare systems, requiring a nuanced approach that bridges the gap between physical and psychological medicine. This review has highlighted the complexities involved in diagnosing and treating SSDs, emphasizing the critical need for collaboration between family medicine and psychiatry.

The evolution of diagnostic criteria for SSDs, as reflected in the DSM‐5, marks a shift towards a more holistic understanding of these disorders. By focusing on the patient's thoughts, feelings, and behaviors in response to their symptoms, rather than solely on the presence of medically unexplained symptoms, clinicians can better capture the essence of these conditions. However, diagnostic challenges persist, particularly in distinguishing SSDs from organic pathology and addressing cultural variations in symptom presentation.

Management strategies for SSDs have shown promising results, especially when combining psychotherapeutic interventions, judicious use of pharmacological treatments, and lifestyle modifications. Cognitive Behavioral Therapy (CBT) and other evidence‐based psychotherapies have emerged as cornerstone treatments, while integrated care approaches offer a framework for comprehensive patient management.

The collaboration between family medicine and psychiatry is paramount in providing optimal care for patients with SSDs. Various models of collaboration, from consultation‐liaison psychiatry to integrated care teams, offer pathways to leverage the expertise of both disciplines. However, challenges such as time constraints and differing practice cultures necessitate ongoing efforts to enhance interdisciplinary cooperation.

Effective communication strategies play a crucial role in managing SSDs, requiring a delicate balance between validating patients' experiences and addressing underlying psychiatric issues. Techniques such as active listening, biopsychosocial framing, and culturally sensitive approaches can significantly improve patient engagement and treatment outcomes.

As our understanding of SSDs continues to evolve, several areas warrant further research and development:

1. Refinement of diagnostic tools and criteria to improve accuracy and cultural sensitivity.
2. Development and evaluation of novel treatment approaches, particularly for treatment‐resistant cases.
3. Implementation and assessment of integrated care models in diverse healthcare settings.
4. Enhancement of communication skills training programs specific to SSD management.

In conclusion, the effective management of somatic symptom disorders requires a multifaceted approach that integrates the expertise of family medicine and psychiatry. By fostering collaboration, employing evidence‐based treatments, and utilizing skilled communication strategies, healthcare providers can improve outcomes for patients with SSDs. As we move forward, continued research and interdisciplinary cooperation will be essential in advancing our ability to address these complex and challenging disorders.

## CONFLICT OF INTEREST STATEMENT

The authors declare no conflict of interest.

## ETHICS STATEMENT

None.
